# Design and utilisation of a novel, high-fidelity, low-cost, hybrid-tissue simulation model to facilitate training in robot-assisted partial nephrectomy

**DOI:** 10.1007/s11701-024-01857-2

**Published:** 2024-03-01

**Authors:** Stefanie M. Croghan, Miroslav Voborsky, Adam F. Roche, Claire Condron, Dara A. O’Keeffe, Barry B. McGuire

**Affiliations:** 1 Department of Urology and Transplantation, Beaumont Hospital, Dublin, Ireland; 2 Strategic Academic Recruitment Programme, Royal College of Surgeons in Ireland, Dublin, Ireland; 3 RCSI SIM Centre for Simulation Education and Research, RCSI University of Medicine and Health Sciences, Dublin, Ireland; 4 Department of Urology, St. Vincent’s University Hospital, Dublin, Ireland

**Keywords:** Simulation, Model, Design, Robotic surgery, Training, Partial nephrectomy

## Abstract

Robot-assisted partial nephrectomy (RAPN) has rapidly evolved as the standard of care for appropriately selected renal tumours, offering key patient benefits over radical nephrectomy or open surgical approaches. Accordingly, RAPN is a key competency that urology trainees wishing to treat kidney cancer must master. Training in robotic surgery is subject to numerous challenges, and simulation has been established as valuable step in the robotic learning curve. However, simulation models are often both expensive and suboptimal in fidelity. This means that the number of practice repetitions for a trainee may limited by cost restraints, and that trainees may struggle to reconcile the skills obtained in the simulation laboratory with real-world practice in the operating room. We have developed a high-fidelity, low-cost, customizable model for RAPN simulation based on porcine tissue. The model has been utilised in teaching courses at our institution, confirming both feasibility of use and high user acceptability. We share the design of our model in this proof-of-concept report.

**Supplementary Information:**

The online version contains supplementary material available at 10.1007/s11701-024-01857-2.

Partial nephrectomy (PN) has become the standard of care for T1 kidney tumours, and may be used in larger tumours in anatomically/functionally solitary kidneys or in CKD, where technically feasible [1]. Minimally invasive PN is associated with decreased morbidity versus open PN, with comparable oncological outcomes [1]. Robot-assisted partial nephrectomy (RAPN) affords the surgeon enhanced dexterity and has been associated with reduced blood loss, decreased warm ischaemia time and lower conversion rates to open or radical procedures, compared to laparoscopic approaches [2, 3]. Unsurprisingly, 10-year US operative trend figures have shown exponential growth in RAPN [4]. It is clearly evident that, where resources allow, RAPN has become the surgical treatment of choice for the small renal mass, and therefore should be a central focus of training in urological oncology.

Surgical training in robotics can be hampered by a number of challenges when delivered intraoperatively, including time constraints and safety concerns regarding relinquishing console control to an inexperienced trainee [5]. Partial nephrectomy is a particularly challenging operation to teach, given pressures to minimise warm-ischaemia time, to achieve a negative resection margin and to minimise complications such as urine leak. Simulation training in robotics has emerged as a highly acceptable modality, proven to enhance subsequent operating room (OR) performance, and is an integral part of evolving robotic surgical curricula [6]. However, a major difficulty with simulation is the provision of an authentic operative experience that will ensure translatability of acquired skills to the OR. Virtual reality programmes are easy to deliver via console simulators, standardized, reproducible and effective [7], and have been successfully designed for RAPN [8]. However, they have significant limitations, notably cost and the inability to replicate realistic tissue handling, leaving surgeons with a wide learning gap when transitioning to real-world practice. Impressive synthetic models have also been utilised for simulation of RAPN [9], but can be costly and may struggle to replicate native tissue texture.

We aimed to design a reproducible, high-fidelity, low-cost, partial nephrectomy simulation model for use during robotic training, based on porcine tissue. This was done via a research-practitioner collaborative steering group, with input from simulation technicians, clinical educators with expertise in simulation, a consultant robotic urological surgeon and a urology trainee. The principles of design-based research were applied [10]. In an iterative process, multiple refinements were made to the model based on surgeon feedback, with the overarching objectives of achieving functional fidelity [11] and reproducibility. The final model underwent pilot testing by a robotic surgical expert, and team consensus was then reached regarding its suitability for use in robotic training.

The model was created using porcine kidneys obtained from 70 kg adult Landrace pigs. The kidneys were provided from our agricultural suppliers, and received *en bloc* with perihilar tissue and ureters remaining intact. We used fresh porcine tissue in this model, but have found cryopreservation at − 20 °C following harvest, with thawing prior to use, to be acceptable also.

The tumour mass itself was modelled in the first instance, with three variations of tumour model designed.

*Synthetic*

The first, synthetic model was created using platinum silicone (Ecoflex™ 00-31 Near Clear™), mixed with Smooth-On Silicone Thinner™ to reduce viscosity, to produce an approximately 3 cm diameter spherical mass. This was then coated with a thin layer of transparent silicone (Ecoflex™ 00-31) to enhance structural integrity and to simulate an encapsulated tumour.

*Biological (Gonad)*

The second tumour model was biological, and used a single harvested bovine ovary as a tumour mass.

*Biological (Thymus)*

The third model used a harvested porcine thymus to simulate a tumour mass.

The porcine kidneys were prepared with minimal disruption of perihilar tissue to allow the trainee the opportunity to robotically dissect the renal hilum, if desired. The ureter was identified and preserved. Using a sharp dissecting scissors, the renal capsule was opened distant from the intended site of tumour location, and separated from the relevant pole of the kidney with blunt dissection. The underlying cortex was demarcated with a surgical marker, representative of the size and configuration of the model tumour. An appropriately sized section of renal cortex was excised (Supp. Figure 1). Two to three stab incisions created communications with the collecting system, to simulate bleeding points. The ureter was cannulated distally with tubing (2 mm x 140 mm Simulated Synthetic Artery; Limbs & Things™), advanced to the renal pelvis, and connected to an intravenous giving set, containing 250 ml NaCl 0.9% coloured with simulated blood powder (Adam,Rouilly™). The irrigation system was opened and the model ‘tumour bed’ inspected to ensure adequate simulation of bleeding (Supp. Video 1). The chosen tumour model was then placed within the cortical defect (Supp. Figure 2), and secured using tissue glue 3M™ Vetbond Tissue Adhesive™ around its perimeter, with avoidance of the intended bleeding sites. Thereafter, the renal capsule was replaced over the tumour-bearing kidney and secured with the same tissue glue. The final model is illustrated in Fig. 1. More than one simulated tumour was placed in each kidney, where desired. The model was placed on a section of porcine abdominal wall, containing musculature to mimic the in vivo environment (Supp. Figure 3), within a laparoscopic box trainer in which ports were placed. A surgical robot was docked, allowing trainees to perform RAPN on the model using the robotic console (Supp. Video 1; Supp. Figure 4).

**Fig. 1:**
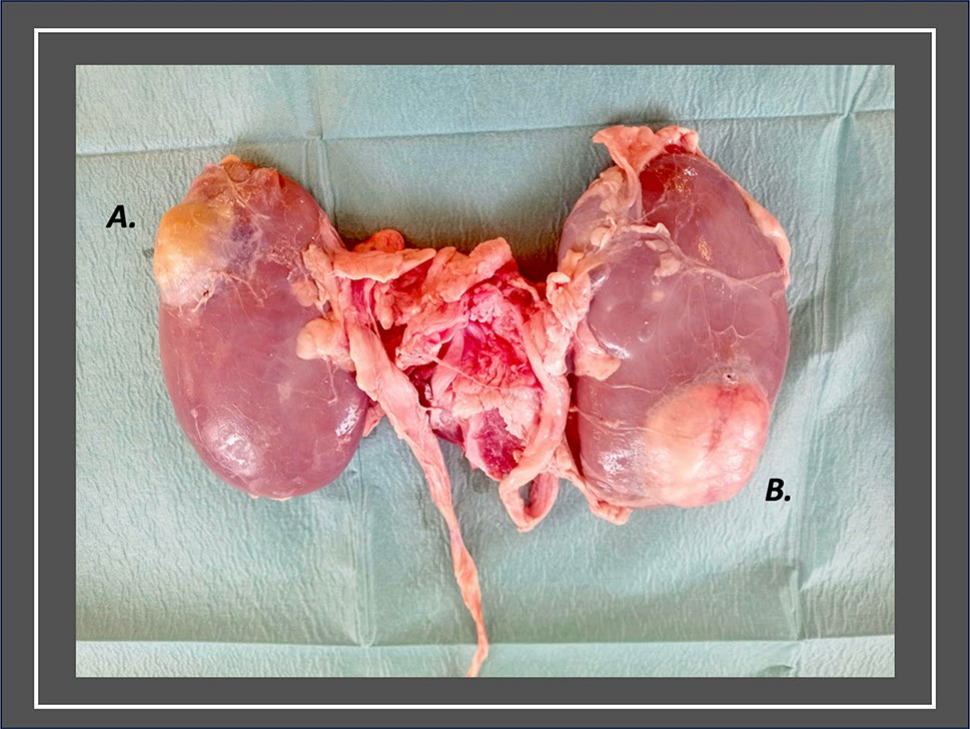
Final RAPN Simulation Model. This figure presents the final porcine simulation model for robot-assisted partial nephrectomy incorporating a silicone model tumour (**A**) and a model tumour derived from bovine gonadal tissue (**B**)

Our model was recently utilised by eight urology trainees/residents and three consultant robotic surgeons during a robotic training day using the Da Vinci Xi (Intuitive Surgical, Sunnyvale, California). All participants were familiar with the procedure of RAPN, and had observed or participated in > 5 cases in real life. Feasibility was confirmed, with all procedures completed as intended. Participants gave feedback via an anonymous questionnaire enquiring about perceptions of the simulator and its potential utility. Models were labelled A (synthetic), B (gonadal) and C (thymic), without disclosure of the material used. Overall, good user acceptability was evident. Face validity was higher with biological tumour models than the synthetic one. Trainees were asked to rank the performance of each model in simulating a real human kidney with a tumour mass on a 10-point scale, where 0 represented ‘very inadequate’ and 10 represented ‘perfect.’ Model A (synthetic) received a mean score of 6, whilst the biological models scored 7.5—8. Trainees (*n* = 8) reported all models to be “satisfactory” or “very satisfactory” in performing tumour dissection and in performing renorrhaphy, with the exception of one respondent who found the dissection with the synthetic model to be unsatisfactory. Free-text comments elaborated to explain that the synthetic tumour enucleated too easily. Content validity was further confirmed with trainees scoring the utility of the models in practicing RAPN (10-point scale) as mean 6 (synthetic) and mean 8–9 (biological). Faculty (*n* = 3) all described the models as “excellent.”

There are a number of major advantages to this simulation model. Firstly, it is low-cost, averaging €50 ($54.75) per unit in our centre. This is a fraction of the cost of other RAPN models [9], and we hope will broaden access and/or facilitate a greater number of practice repetitions per trainee. The model is anatomically accurate, and allows trainees the opportunity to complete major steps in robotic partial nephrectomy, including clamping of renal vessels, tumour excision and renorrhaphy, whilst providing a realistic tissue-handling experience with the impression of active bleeding. The latter replicates the generalised bleeding that often occurs during RAPN, despite vascular clamping and preparation. This creates time pressure and obscures vision, making the dissection plane difficult to appreciate, causing stress to the surgeon, and potentially risking positive tumour margins. Simulation of this allows the addition of an added layer of pressure, representative of that which might be encountered in the OR, after a trainee has demonstrated competency in the basic procedural steps. Our impression is that the porcine tissue provides a higher fidelity experience than alternatives such as hydrogel models, with more realistic haptic feedback and behaviour regarding suture passage and diathermy. Furthermore, the flexibility of design allows customisation of the model according to users’ level of skill and learning objectives. Using the R.E.N.A.L nephrometry score [12] as a guide, a bespoke simulation can be created with variations on tumour size, endophytic/exophytic nature, location relative to the collecting system, and position within the kidney. We therefore have wide scope to create authentic simulations of high-complexity RAPN, with standardization, for surgeons seeking a greater challenge. In future iterations, we plan to create a fully endophytic tumour model for use with intraoperative ultrasonographic tumour location.

There are, of course, some potential limitations to our model. Clearly, a training centre requires the use of a surgical robot to best utilise this model, and the low-cost nature of the simulation is dependent on pre-existing access to the surgical robot itself. Our initial evaluation suggests a preference of trainees for the biological tumour simulation as opposed to the synthetic one. This was largely due to the tissue glue used adhering less effectively to the synthetic material, meaning that the silicone tumour could be easily enucleated in some cases, limiting sharp dissection. Whilst trainees also expressed preference for the texture of the biological models, faculty observed that the silicone model did mimic the texture of more gelatinous renal masses (e.g. angiomyolipomas or cystic renal cell carcinomas), and may therefore retain an important role. We do acknowledge that access to animal tissue may vary between training centres, or may not be acceptable to all users. Nonetheless, we feel that this model could have wide applicability and be readily adopted by numerous other surgical training centres. We acknowledge the need for further objective evaluation data centred on skills acquisition and trainee performance improvement, and plan to obtain this in a new robotic training curriculum currently under development at our institution.

In summary, we propose a novel, porcine-derived, hybrid-tissue design that provides a cost-effective, high-fidelity simulation model for use in the training of partial nephrectomy. Our model is readily customisable, has demonstrated high levels of acceptability to both robotic trainers and trainees, and could be adopted by other training centres with ease. Further testing will be required to established this models relationships with associated variables.

## Supplementary Information

Below is the link to the electronic supplementary material.

**Supplementary Figure 1 – Excision of Native Parenchyma**. This figure demonstrates dissection of the renal capsule from the kidney parenchyma and excision of a portion of cortex for model tumour placement. (JPEG 614 kb)

**Supplementary Figure 2 - Tumour Placement**. This figure shows the placement of a silicone tumour model into the parenchymal cavity created. It is subsequently glued in place and covered over with the capsule. (JPEG 644 kb)

**Supplementary Figure 3 – Presentation of Model**. Here we show the final presentation of the tumour-bearing models placed on porcine abdominal wall musculature. A = biological tumour. B = synthetic tumour. C = arterial tubing for inflow of simulated blood. (PNG 5033 kb)

**Supplementary Figure 4**. Trainee performing robotic renorrhaphy on the simulation model following tumour excision (PNG 3948 kb)

**Supplementary Video 1**: RAPN Model. This video demonstrates the design and utilisation of our RAPN simulation model, with footage of simulated bleeding, robotic tumour dissection and renorrhaphy. Music credit: Nature by MaxKoMusic (https://maxkomusic.com); promoted by https://www.chosic.com/free-music/all. Creative Commons Attribution-ShareAlike 3.0 Unported. (MP4 174783 kb)

## Data Availability

No datasets were generated or analysed during the current study.
