# Supplementary figures and images for: Design and utilisation of a novel, high-fidelity, low-cost, hybrid-tissue simulation model to facilitate training in robot-assisted partial nephrectomy
Source: J Robot Surg. 2024 Mar 1;18(1):103. doi: 10.1007/s11701-024-01857-2 (PMC10907476; doi:10.1007/s11701-024-01857-2)

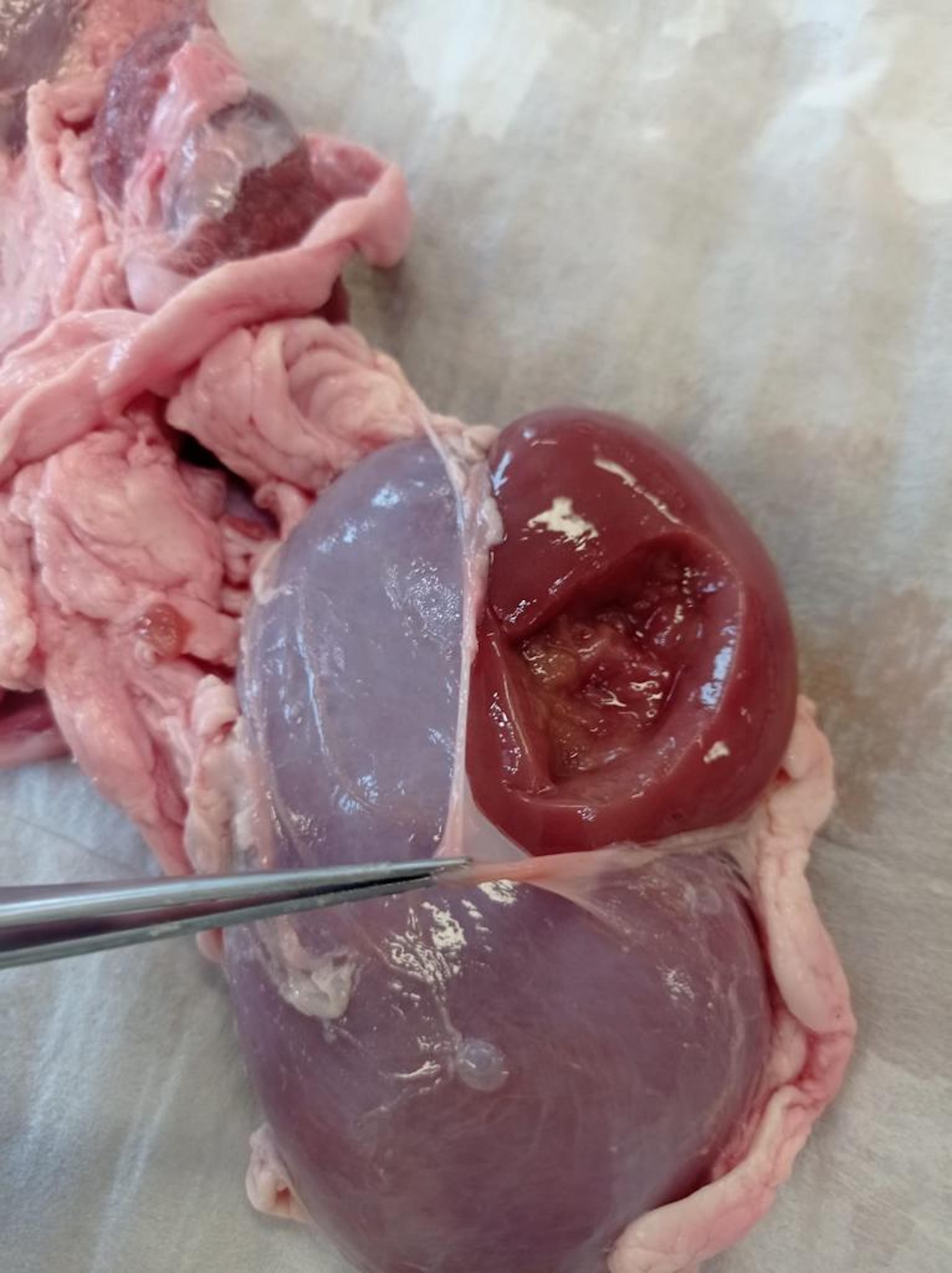

Supplement: Supplementary file 1 — Supplementary Figure 1 – Excision of Native Parenchyma. This figure demonstrates dissection of the renal capsule from the kidney parenchyma and excision of a portion of cortex for model tumour placement. (JPEG 614 kb) [file 11701_2024_1857_MOESM1_ESM.jpeg]

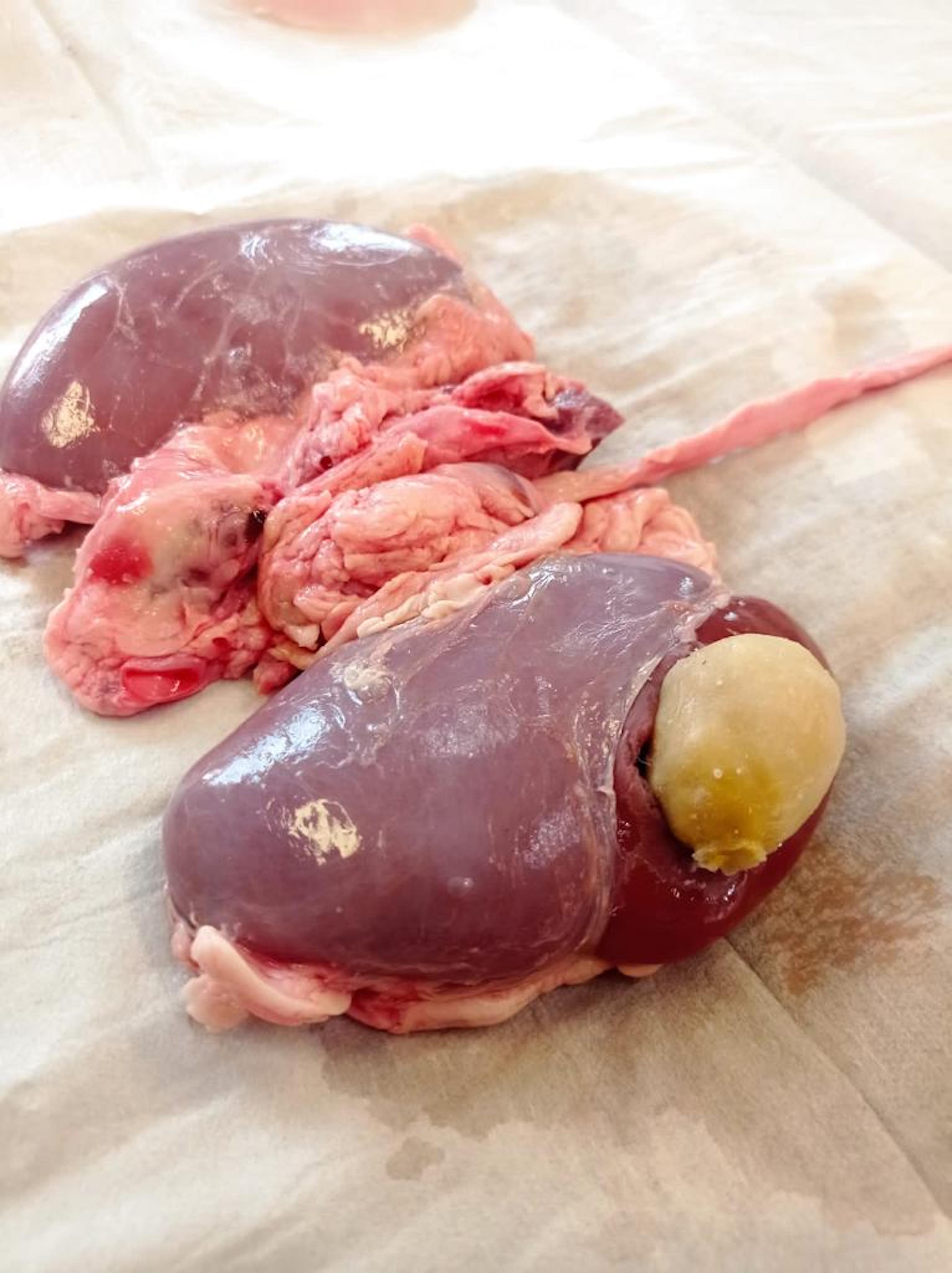

Supplement: Supplementary file 2 — Supplementary Figure 2 - Tumour Placement. This figure shows the placement of a silicone tumour model into the parenchymal cavity created. It is subsequently glued in place and covered over with the capsule. (JPEG 644 kb) [file 11701_2024_1857_MOESM2_ESM.jpeg]

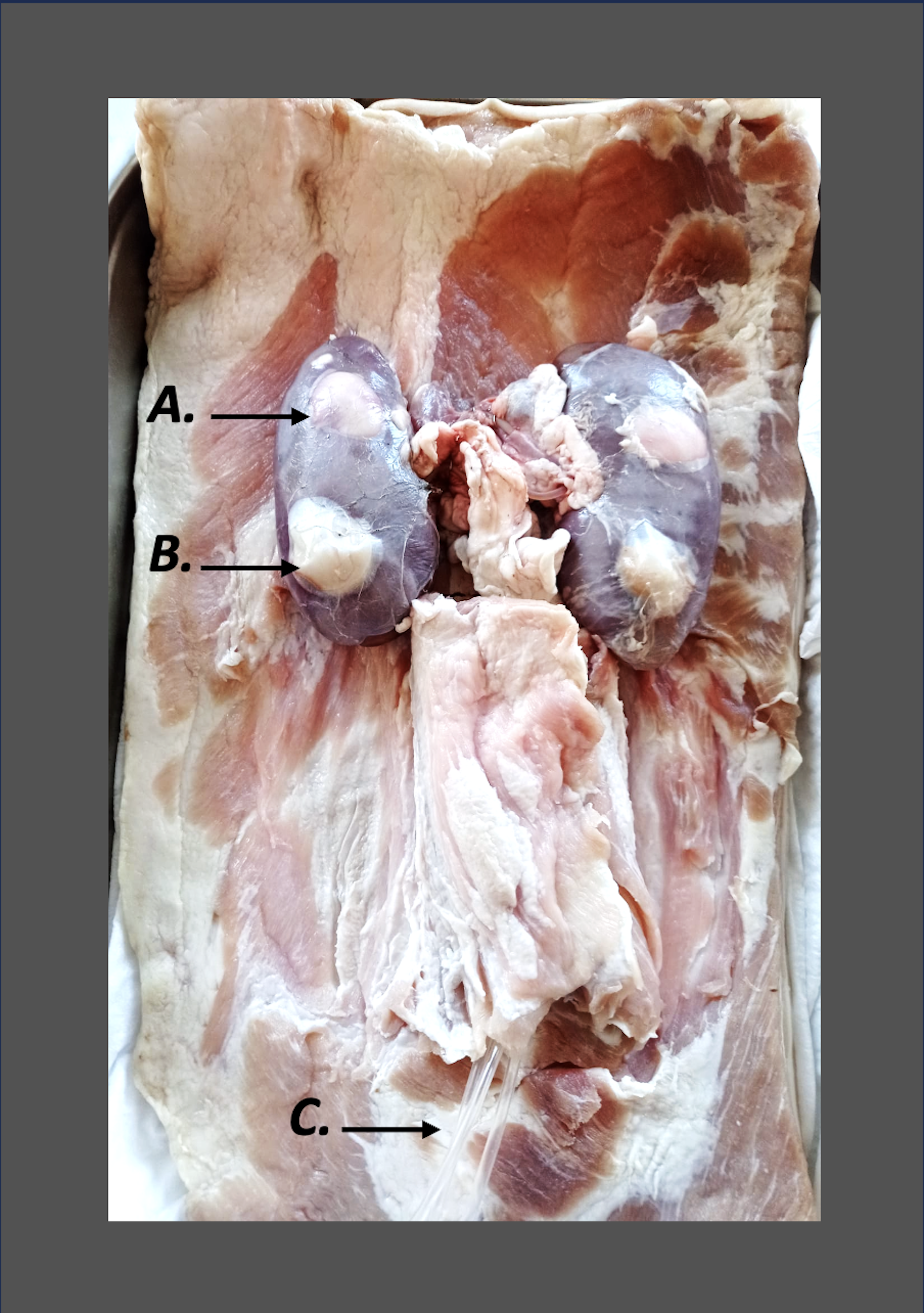

Supplement: Supplementary file 3 — Supplementary Figure 3 – Presentation of Model. Here we show the final presentation of the tumour-bearing models placed on porcine abdominal wall musculature. A = biological tumour. B = synthetic tumour. C = arterial tubing for inflow of simulated blood. (PNG 5033 kb) [file 11701_2024_1857_MOESM3_ESM.png]

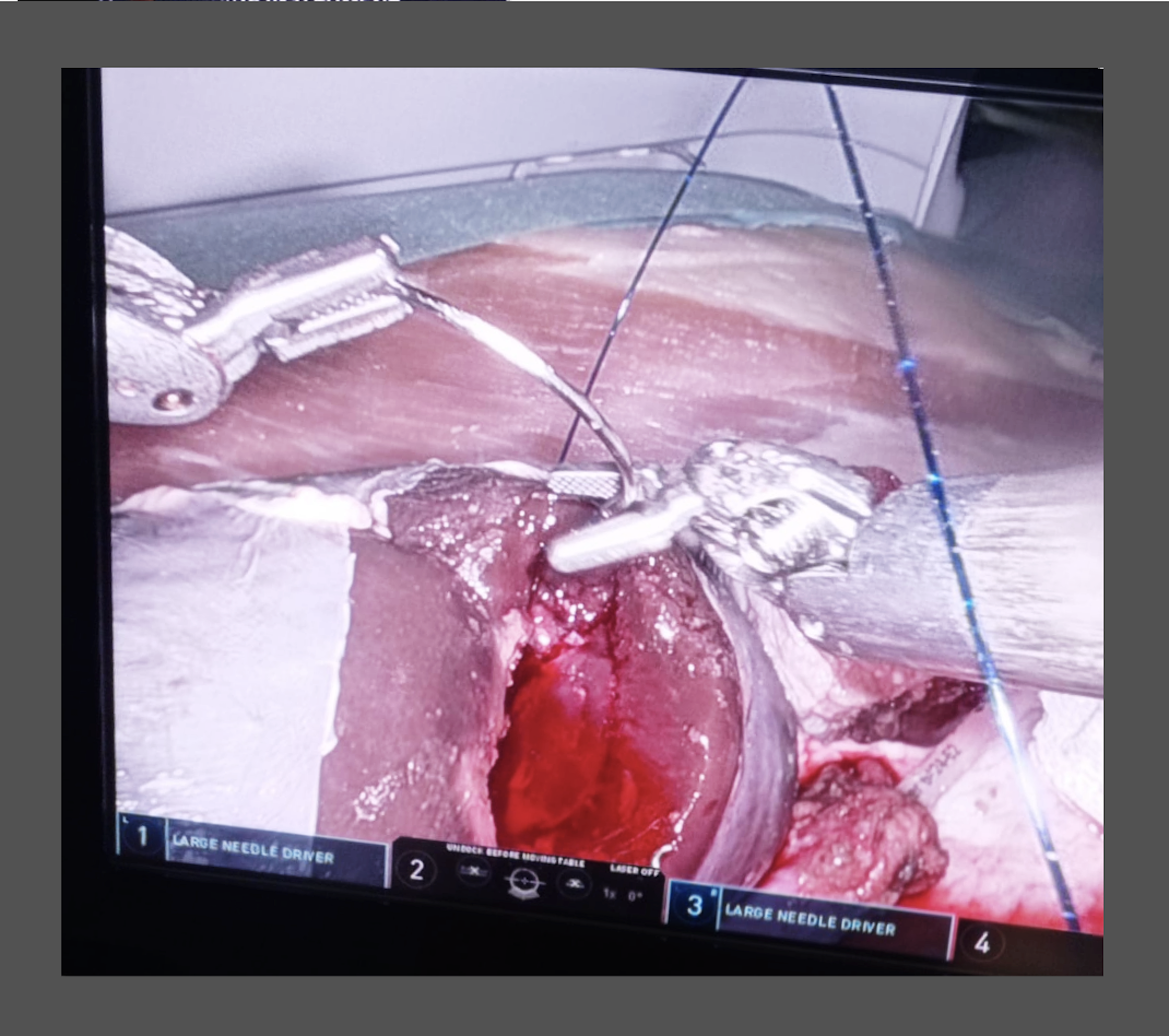

Supplement: Supplementary file 4 — Supplementary Figure 4. Trainee performing robotic renorrhaphy on the simulation model following tumour excision (PNG 3948 kb) [file 11701_2024_1857_MOESM4_ESM.png]
